# Modified triglyceride-glucose indices as novel predictors of metabolic dysfunction-associated fatty liver disease in US adolescents: a nationally representative study from NHANES 2017–2020

**DOI:** 10.1186/s12876-025-03915-x

**Published:** 2025-05-01

**Authors:** Yigui Zou, Yu Dai, Ziyuan Li, Baixian Lin, Hu Chen, Zeling Zhuang, Wenwen Li, Qinghua Yang, Dongling Dai

**Affiliations:** 1 Key Laboratory for Precision Diagnosis and Treatment of Pediatric Digestive System Disease, Endoscopy Center, Shenzhen Children’s Hospital, Shenzhen, Guangdong, 518036 China; 2 Children’s Healthcare and Mental Health Center, Shenzhen Children’s Hospital, Shenzhen , Guangdong, 518036 China

**Keywords:** TyG index, Metabolic dysfunction-associated fatty liver disease, Insulin resistance, Adolescent, NHANES

## Abstract

**Background:**

Metabolic dysfunction-associated fatty liver disease (MAFLD) has become the most prevalent chronic liver condition in adolescents. The triglyceride-glucose (TyG) index, a surrogate marker of insulin resistance, has shown promise in adult MAFLD detection but requires pediatric-specific validation, particularly when combined with anthropometric measures. This study investigated the association between modified TyG indices and MAFLD, and evaluated their predictive value in adolescents.

**Methods:**

This cross-sectional study analyzed data from 532 adolescents (12–18 years) in the 2017–2020 National Health and Nutrition Examination Survey (NHANES) with complete records. MAFLD diagnosis was based on transient elastography plus metabolic criteria. The investigators employed multivariate linear regression and restricted cubic splines (RCS) to examine linear and nonlinear relationships between modified TyG indices and CAP values. Subgroup analyses were stratified by obesity status, and sensitivity analyses were performed on the NAFLD cohort (n = 527). Receiver operating characteristic (ROC) curve analysis, using Youden's index, evaluated the predictive performance of TyG indices for MAFLD identification.

**Results:**

Among 130 MAFLD adolescents (vs 402 controls), modified TyG indices demonstrated significantly stronger associations with CAP in fully adjusted models compared to the original TyG index. TyG-WC showed the highest diagnostic accuracy (AUC = 0.923, 95%CI:0.900–0.947), followed by TyG-BMI (AUC = 0.917) and TyG-WHtR (AUC = 0.915), while the original TyG index performed poorly (AUC = 0.673). Subgroup analyses revealed particularly strong associations in non-obese participants, and sensitivity analyses confirmed result robustness after excluding potential confounders. Optimal cutoff values provided clinically useful screening thresholds, with TyG-WC achieving 94% sensitivity at 665.94.

**Conclusion:**

This study demonstrates that modified TyG indices incorporating anthropometric parameters (particularly TyG-WC) significantly outperform the original TyG index for MAFLD detection in adolescents, with superior diagnostic accuracy (AUC 0.915–0.923). The robust predictive performance maintained in sensitivity analyses and non-obese subgroups supports their clinical utility as simple, non-invasive screening tools for pediatric MAFLD risk stratification.

## Background

Non-alcoholic fatty liver disease (NAFLD) is a chronic liver disease characterized by accumulation of excessive fat in hepatocytes in the absence of significant alcohol consumption. The disease spectrum encompasses a wide range of pathological stages, from simple steatosis and non-alcoholic steatohepatitis (NASH) to hepatic fibrosis and hepatocellular carcinoma (HCC). Globally, the prevalence of NAFLD is estimated at 30.2% in the general population [1], with approximately 13% among adolescent [2]. Moreover, NAFLD is associated with obesity, type 2 diabetes [3], and cardiovascular disease, contributing to a significant public health and economic burden [4].

Recently, a panel of interntional experts proposed redefining NAFLD as ‘metabolic associated fatty liver disease’ (MAFLD) to better reflect its underlying disease pathology and strong association with metabolic comorbidities. Unlike NAFLD, the diagnosis of MAFLD does not exclude alcohol consumption or the coexistence of other liver diseases, such as viral hepatitis. The proposed diagnostic criteria for MAFLD require evidence of hepatic steatosis, confirmed by histology, imaging, or blood biomarkers, in combination with at least one of the following metabolic abnormalities: overweight/obesity, type 2 diabetes mellitus (T2DM), or metabolic dysregulation [5]. The global prevalence of MAFLD is substantial, with estimates of 39.22% in the general population [6], 50.7% among overweight/obese adults [7].

The pathogenesis of MAFLD remains incompletely understood. However, increased hepatic triglyceride accumulation and insulin resistance (IR) are critical in the disease's onset [8]. IR is characterized by impaired suppression of lipolysis and excess fat accumulation through adipocyte de novo lipogenesis in the presence of elevated insulin serum levels [9]. The TyG index, derived from fasting triglyceride and glucose levels, has emerged as a reliable surrogate marker of IR, particularly for peripheral and hepatic IR. Evidence suggests that the TyG index is not only associated with IR but also serves as a predictor for diabetes, cardiovascular diseases, and NAFLD [10, 11]. Furthermore, elevated TyG index levels have been linked to increased risks of all-cause and cardiovascular mortality [12]. While the TyG index has demonstrated a strong association with NAFLD in adult populations [13–15], its relationship with pediatric MAFLD remains underexplored and warrants further investigation,, particularly under the revised diagnostic criteria.

Liver biopsy remains the gold standard diagnostic tool for MAFLD, yet its invasiveness and complications, such as internal bleeding and abdominal pain, limit its application in pediatric populations. Consequently, there is an urgent need for non-invasive and effective diagnostic tools. Recent studies [16–21] have highlighted the potential of various obesity indices, including body mass index (BMI), waist circumference (WC), waist-to-hip ratio (WHR), and waist-to-height ratio (WHtR), as predictors of NAFLD.

Therefore, this study aimed to evaluate both the association and predictive value of the original TyG index and its anthropometric-enhanced variants (TyG-BMI, TyG-WC, TyG-WHR, TyG-WHtR) for MAFLD identification in a nationally representative adolescent cohort.

## Methods

### Data sources and study population

The National Health and Nutrition Examination Survey (NHANES), conducted by the National Center for Health Statistics (NCHS), is a nationally representative survey designed to assess the health and nutritional status of both adults and children in the United States. NHANES collects demographic and in-depth health information through home visits, screening, and laboratory testing conducted at a mobile examination center (MEC). Written informed consent was obtained from all participants prior to their inclusion in the survey. As this study involved secondary analysis of publicly available NHANES data, additional Institutional Review Board (IRB) approval was not required. The NHANES data are available via the NHANES website (http://www.cdc.gov/nchs/nhanes.htm).

**Fig. 1:**
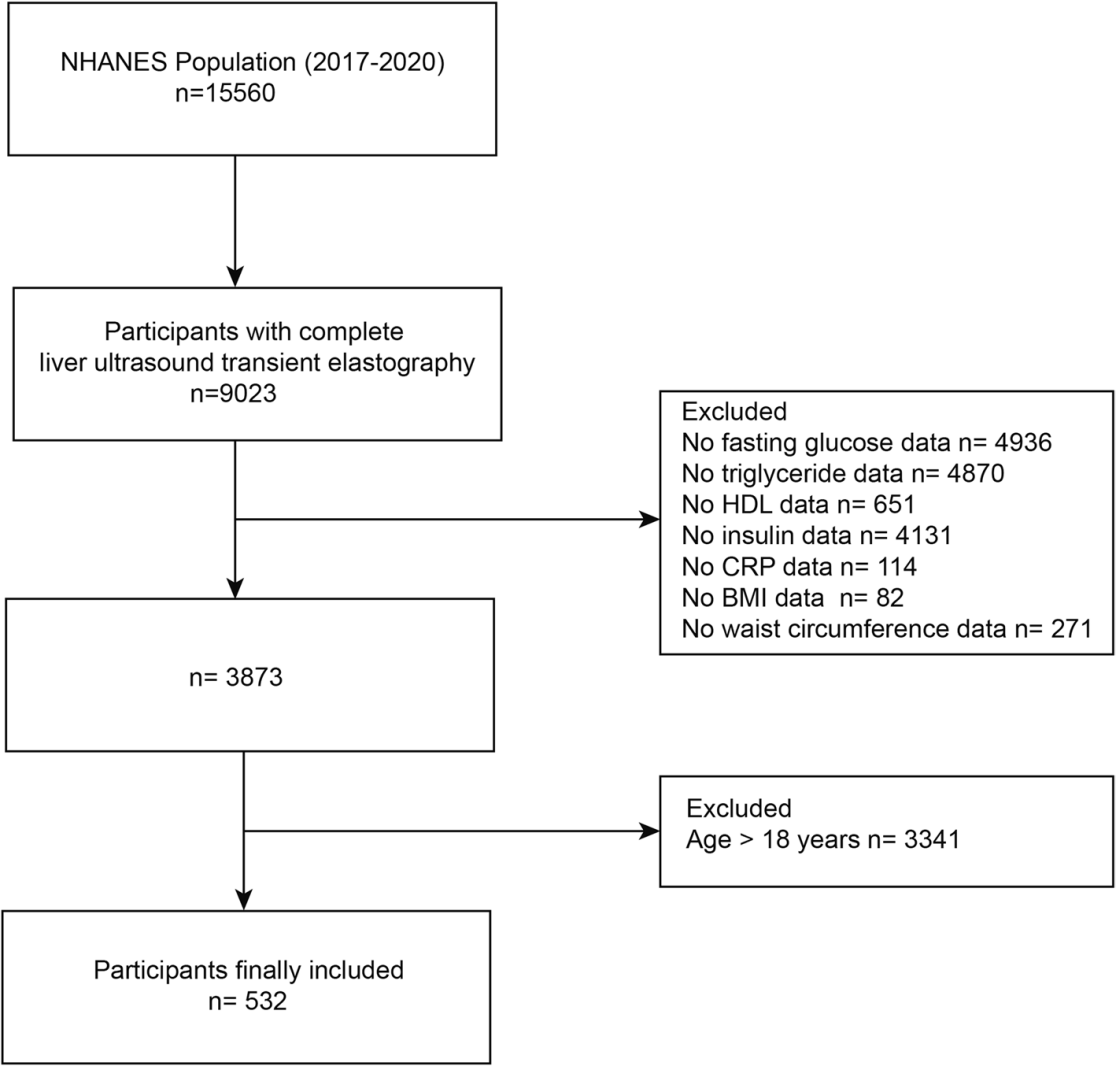
Flow diagram of participants. NHANES, National Health and Nutrition Examination Survey

In our research, data from 2017–2020 NHANES cycles were utilized, and all details were retrieved from the official website. We included adolescent (≤ 18 years) with complete data on liver ultrasound transient elastography, fasting glucose, triglyceride, high-density lipoprotein(HDL), C-reactive protein (CRP), body mass index (BMI), and waist circumference (WC) datas. A total of 532 participants were included according to the MAFLD diagnostic criteria (Fig. 1).

### Hepatic steatosis

Hepatic steatosis was detected by vibration-controlled transient elastography (VCTE). In the 2017–2018 and 2019–2020 cycle, VCTE was performed by trained technicians using the FibroScan® model 502 V2 Touch device equipped with medium (M) or extra-large (XL) probes. All participants aged 12 years and older were eligible unless they met any of the following exclusion criteria: inability to lie flat, current pregnancy, presence of an implanted electronic medical device, or skin lesions at the measurement site. To ensure data quality, only participants meeting the following criteria were included in the analysis: a fasting time of at least 3 h, a minimum of 10 valid stiffness (E) measurements, and a liver stiffness interquartile range/median < 30%. Hepatic steatosis was defined as a median controlled attenuation parameter (CAP) score ≥ 248 dB/m, based on established diagnostic thresholds [22].

### Diagnosis of MAFLD

MAFLD was diagnosed in the presence of steatosis (evaluated by CAP) plus at least one of the following criteria: overweight or obesity, prediabetes or diabetes, and at least two metabolic abnormalities [5, 23]. Metabolic abnormalities included elevated BP, triglyceride levels ≥ 150 mg/dl, high-density lipoprotein (HDL) cholesterol levels < 40 mg/dL, and triglycerides-to-HDL cholesterol ratio > 2.25 (while adult MAFLD criteria were applied for adolescents 16 years and older).

### TyG index

Blood samples for glucose and triglyceride measurements were collected during the mobile examination center (MEC) visits. These samples were processed, stored at − 30 °C, and subsequently shipped to the University of Missouri-Columbia and the University of Minnesota for analysis. Detailed procedures for specimen collection, handling, and processing are documented in the NHANES Laboratory/Medical Technician Procedures Manual (LPM). Triglyceride to glucose index was calculated using the formula: TyG index = Ln [fasting triglycerides (mg/dL) × fasting glucose (mg/dL)]/2[24]. Modified TyG indices were calculated as follows: TyG-BMI = TyG × BMI; TyG-WC = TyG × WC; TyG-WHR = TyG × WHR; TyG-WHtR = TyG × WHtR [14, 25, 26].

### Other covariates

The study included demographic, anthropometric, and laboratory variables as covariates. Demographic variables included age, gender, and race/ethnicity (categorized as Mexican American, other Hispanic, non-Hispanic White, non-Hispanic Black, and Other [including multiracial]). Anthropometric measures included height, weight, body mass index (BMI), waist circumference (WC), hip circumference, and blood pressure. BMI was calculated as weight (kg) divided by height (m^2^) and categorized as underweight, normal weight, overweight, and obese. Hypertension was defined as systolic blood pressure (SBP) > 130 mmHg or diastolic blood pressure (DBP) > 85 mmHg. Laboratory data included aspartate aminotransferase (AST), alanine aminotransferase (ALT), glycohemoglobin (HbA1c), insulin, total cholesterol (TC), low-density lipoprotein (LDL), high-density lipoprotein (HDL), C-reactive protein (CRP), and total bilirubin. Diabetes was defined as HbA1c ≥ 6.5% or a self-reported diagnosis, while prediabetes was defined as HbA1c between 5.7% and 6.5% [27] or a self-reported diagnosis of prediabetes.

### Statistical analysis

Continuous variables were expressed as weighted means ± standard error (SE), and categorical variables were summarized as weighted proportions. Group comparisons were performed using the t-test for continuous variables (assuming normal distribution) and the chi-square (χ^2^) test for categorical variables. To further analyze the relationship between TyG indices and MAFLD, univariate and multivariate liner regression models were employed.

Multivariate analyses included: (1) unadjusted models, (2) model 1 adjusted covariates including age, gender, and race, and (3) fully adjusted models. To evaluate the potential nonlinear relationship between TyG indices and MAFLD, restricted cubic spline (RCS) regression was performed. Four nodes located in the 5 th, 35 th, 65 th, and 95 th percentiles of different TyG index are used in restricted cubic spline. The significance of nonlinearity was tested using the spline test.

Receiver operating characteristic (ROC) curve analysis was performed to evaluate the diagnostic accuracy of five TyG-related indices for MAFLD detection. The area under the curve (AUC) with 95% confidence intervals (CI) was calculated to assess overall diagnostic performance. Optimal cutoff values were determined by maximizing Youden's index. Corresponding sensitivity, specificity, positive predictive value (PPV), and negative predictive value (NPV) at the optimal cutoffs were computed.

Subgroup analyses were conducted to explore the potential modifying effect of obesity on the association between TyG indices and MAFLD. Sensitivity analyses were conducted by excluding hepatitis B/C-infected (n = 2) and heavy alcohol-consuming participants (*n* = 3), confirming robust TyG-CAP associations in the final NAFLD cohort (n = 527) using identical multivariate models (Models 1–3). All analyses were performed using R version 4.4.2, incorporating appropriate sampling weights as recommended by the National Center for Health Statistics (NCHS). A two-sided p-value < 0.05 was considered statistically significant.

## Results

### Baseline characteristics of the study population

This study enrolled 532 participants, included 276 males and 256 females. Among them, 130 were diagnosed with MAFLD and 402 were non-MAFLD controls. Compared to the non-MAFLD group, patients with MAFLD were predominantly Non-Hispanic white and exhibited significantly higher levels of weight, BMI, waist circumference, AST, ALT, fasting glucose, insulin, LDL, triglyceride, CRP, HOMA-IR, WHR, WHtR, TyG index, TyG-BMI, TyG-WC, TyG-WHR, TyG-WHtR, and CAP, but significantly lower levels of total bilirubin and HDL. Additionally, the majority of MAFLD participants were classified as having prediabetes or diabetes and obesity (Table 1).

**Table 1:** Baseline characteristics of the study population

Variable	Total (*n* = 532)	Non-MAFLD (*n* = 402)	MAFLD (*n* = 130)	*p*
Age (years)	14.98 (0.10)	14.87 (0.12)	15.36 (0.25)	0.064
Gender (%)				0.476
Male	276 (52.0)	208 (51.1)	68 (54.9)	
Female	256 (48.0)	194 (48.9)	62 (45.1)	
Race (%)				0.007
Mexican American	90 (17.0)	59 (13.1)	31 (30.1)	
Other Hispanic	52 (8.2)	34 (7.2)	18 (11.4)	
Non-Hispanic White	169 (50.3)	132 (54.3)	37 (37.0)	
Non-Hispanic Black	119 (13.1)	95 (13.6)	24 (11.6)	
Other Race	102 (11.4)	82 (11.9)	20 (9.8)	
Weight (kg)	66.26 (0.98)	59.81 (0.82)	87.91 (1.75)	< 0.001
Height (cm)	166.03 (0.55)	165.86 (0.67)	166.59 (1.03)	0.307
BMI (kg/m^2^)	23.92 (0.31)	21.65 (0.29)	31.56 (0.59)	< 0.001
Hip circumference (cm)	96.37 (0.60)	92.21 (0.64)	110.35 (1.07)	< 0.001
Waist circumference (cm)	81.58 (0.85)	76.04 (0.58)	100.21 (1.34)	< 0.001
Diastolic blood pressure (mmHg)	64 (0.4)	63 (0.5)	68 (0.9)	< 0.001
Systolic blood pressure (mmHg)	109 (0.6)	108 (0.7)	110 (1.2)	0.212
AST (U/L)	19.70 (0.44)	19.20 (0.52)	21.42 (0.74)	0.009
ALT (U/L)	16.16 (0.48)	14.53 (0.53)	21.66 (1.32)	< 0.001
Total Bilirubin (umol/L)	8.45 (0.35)	8.98 (0.43)	6.66 (0.34)	0.001
HbA1c (%)	5.25 (0.01)	5.24 (0.01)	5.30 (0.03)	0.070
Fasting glucose (mmol/L)	5.41 (0.03)	5.38 (0.03)	5.51 (0.06)	0.019
Insulin (mU/L)	12.86 (0.46)	10.21 (0.40)	21.80 (1.11)	< 0.001
Total cholesterol (mmol/L)	3.96 (0.06)	3.93 (0.07)	4.05 (0.06)	0.082
LDL cholesterol (mmol/L)	2.25 (0.05)	2.21 (0.05)	2.38 (0.04)	0.003
Triglyceride (mmol/L)	0.78 (0.03)	0.73 (0.03)	0.98 (0.05)	< 0.001
HDL cholesterol (mmol/L)	1.36 (0.02)	1.39 (0.02)	1.22 (0.03)	< 0.001
CRP (mg/L)	1.98 (0.15)	1.63 (0.20)	3.17 (0.24)	< 0.001
HOMA-IR	3.13 (0.12)	2.46 (0.10)	5.38 (0.28)	< 0.001
WHtR	0.49 (0.01)	0.46 (0.00)	0.60 (0.01)	< 0.001
WHR	0.84 (0.01)	0.82 (0.00)	0.91 (0.01)	< 0.001
TyG index	8.00 (0.04)	7.94 (0.05)	8.22 (0.05)	< 0.001
TyG-BMI	191.1 (2.84)	171.95 (2.45)	259.92 (5.25)	< 0.001
TyG-WC	654.60 (7.99)	603.99 (5.65)	824.79 (12.14)	< 0.001
TyG-WHtR	3.95 (0.05)	3.64 (0.04)	4.79 (0.08)	< 0.001
TyG-WHR	6.75 (0.06)	6.54 (0.05)	7.46 (0.07)	< 0.001
CAP (db/m)	220.75 (3.44)	198.40 (1.61)	295.91 (4.66)	0.009
Hypertension (%)	15 (3.4)	6 (2.5)	9 (6.6)	0.154
Diabetes or prediabetes (%)	57 (8.5)	31 (6.2)	26 (16.2)	0.002
Obesity (%)	126 (20.9)	34 (6.3)	92 (70.4)	< 0.001

Data are expressed as weighted means ± standard error (SE) for continuous variables and weighted proportions for categorical variables. *BMI* Body Mass Index; *AST* aspartate aminotransferase; *ALT* alanine aminotransferase; *HbA1c* Hemoglobin A1c; *LDL* low density lipoprotein; *HDL* high density lipoprotein; *CRP* C-reactive protein; *HOMA-IR* homeostatic model assessment for insulin resistance; *TyG* triglyceride to glucose index; *WHR* waist-to-hip ratio; *WHtR* waist-to-height ratio; *CAP* controlled attenuation parameter

### Associations of TyG-index and modified TyG-indices with CAP among adolescents

Table 2 shows the results of multivariate linear regression analyses across three models. In unadjusted model and model 2, there was a significantly positive linear association between TyG index, TyG-BMI, TyG-WC, TyG-WHR, TyG-WHtR, and CAP. However, in Model 3, significant positive linear associations persisted for TyG-BMI, TyG-WC, TyG-WHR, and TyG-WHtR, while no significant associations were found for the TyG index.

**Table 2:** Associations of TyG-index and modified TyG-index with CAP among adolescents

Variables	Model1		Model2		Model3	
	β (95%CI)	*P* value	β (95%CI)	*P* value	β (95%CI)	*P* value
TyG index	27.15 (16.06 ~ 38.23)	< 0.001	27.18 (15.07 ~ 39.29)	< 0.001	− 5.04 (− 34.76 ~ 24.68)	0.662
TyG-BMI	0.63 (0.55 ~ 0.70)	< 0.001	0.62 (0.53 ~ 0.72)	< 0.001	0.27 (− 0.10 ~ 0.44)	0.012
TyG-WC	0.26 (0.23 ~ 0.30)	< 0.001	0.26 (0.22 ~ 0.30)	< 0.001	0.15 (0.10 ~ 0.21)	0.002
TyG-WHR	38.24 (30.42 ~ 46.06)	< 0.001	38.75 (30.99 ~ 46.50)	< 0.001	17.05 (6.62 ~ 27.49)	0.011
TyG-WHtR	42.12 (36.73 ~ 47.52)	< 0.001	41.89 (35.14 ~ 48.65)	< 0.001	20.48 (8.29 ~ 32.67)	0.010

*TyG* Triglyceride to glucose index; *BMI* Body Mass Index; *WC* waist circumference; *WHR* waist-to-hip ratio; *WHtR* waist-to-height ratio

Model 1 was unadjusted

Model 2 adjusted for variables including age, gender, and race

Model 3 was fully adjusted

**Fig. 2:**
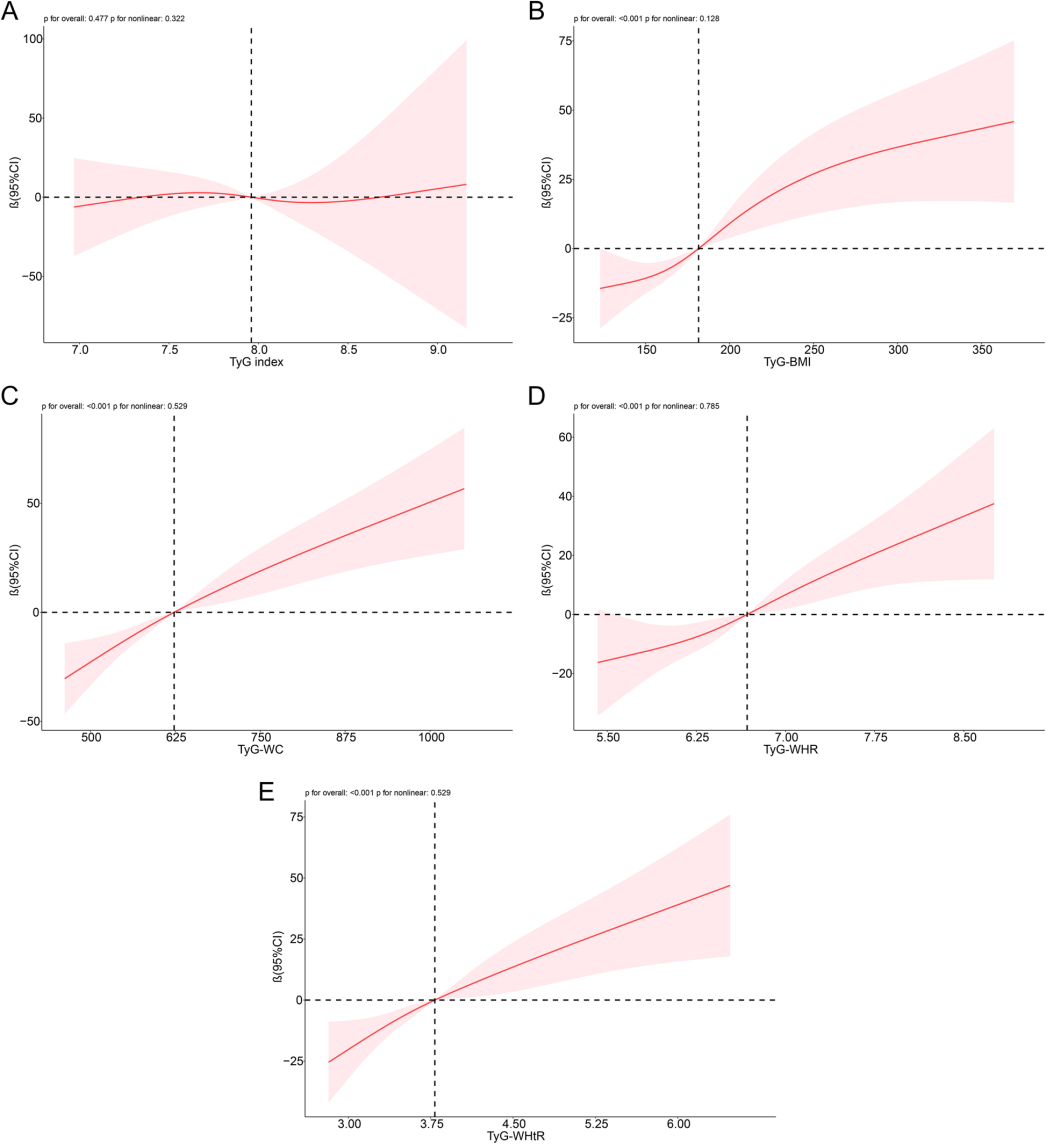
RCS to analyze the nonlinear relationship between TyG index, modified TyG indices and CAP. **A** TyG index; **B** TyG-BMI; **C** TyG-WC; **D** TyG-WHR; **E** TyG-WHtR; TyG index, triglyceride to glucose index; BMI, body mass index; WC, waist circumference; WHR, waist to hip circumference ratio; WHtR, waist to height ratio; 95%Cl, 95% confidence interval

To further explore potential nonlinear relationships, restricted cubic spline (RCS) analyses were performed. The results revealed an S-shape relationship between TyG-BMI and CAP, as well as positive linear relationship between TyG-WC, TyG-WHR, TyG-WHtR and CAP. In contrast, no significant relationship was observed between the TyG index and CAP (Fig. 2).

### Diagnostic performance

**Fig. 3:**
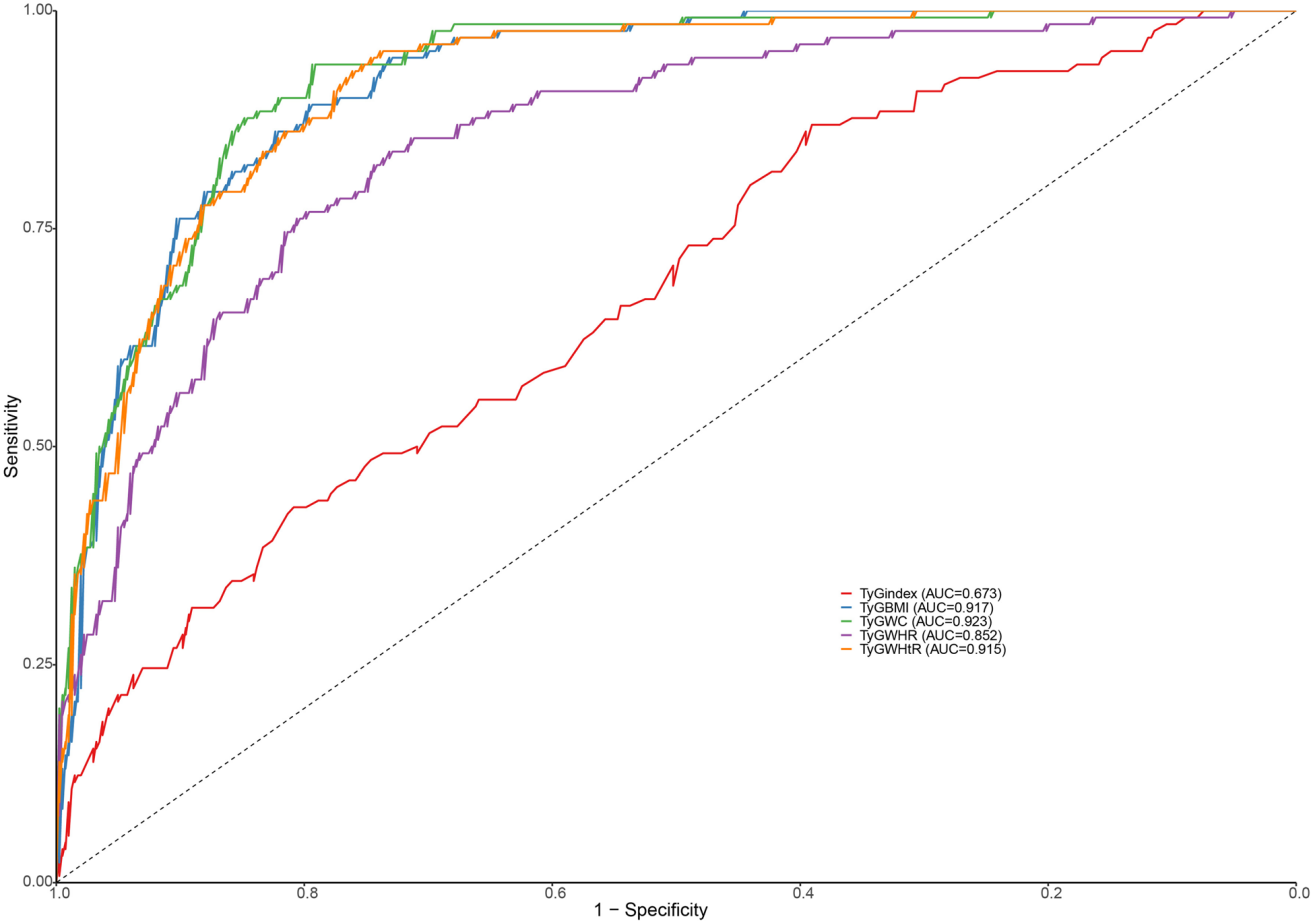
Receiver operating characteristic (ROC) curves of each parameter for predicting MAFLD. TyG, Triglyceride to glucose index; BMI, Body Mass Index; WC, waist circumference; WHR, waist-to-hip ratio; WHtR, waist-to-height ratio

The diagnostic performance of TyG-related indices is presented in Table 3. All five indices demonstrated significant discriminative ability for MAFLD identification (all AUCs > 0.85 except TyG index) (Fig. 3). TyG-WC showed the highest diagnostic accuracy (AUC = 0.923, 95% CI: 0.900–0.947), with optimal cutoff at 665.94 yielding 94% sensitivity and 79% specificity. TyG-BMI and TyG-WHtR also exhibited excellent performance (AUC = 0.917 and 0.915 respectively). Notably, TyG-WHtR at cutoff 3.93 achieved the highest sensitivity (95%) among all indices, while TyG-BMI showed the best balance between sensitivity (89%) and specificity (80%). The original TyG index had the lowest discriminative power (AUC = 0.673), particularly demonstrating low specificity (39%) despite high sensitivity (87%).

**Table 3:** Diagnostic performance of each parameter for predicting MAFLD

Area Under Curve		Cutoff	Diagnostic Performance				
Index	AUC	95% CI	Optimal Cutoff	Sensitivity (%)	Specificity (%)	PPV (%)	NPV (%)
TyG index	0.673	(0.621–0.726)	7.75	0.87	0.39	0.32	0.9
TyG-BMI	0.917	(0.892–0.941)	199.37	0.89	0.8	0.59	0.96
TyG-WC	0.923	(0.900–0.947)	665.94	0.94	0.79	0.6	0.98
TyG-WHR	0.852	(0.815–0.889)	6.83	0.84	0.73	0.5	0.93
TyG-WHtR	0.915	(0.890–0.940)	3.93	0.95	0.74	0.54	0.98

*TyG* Triglyceride to glucose index; *BMI* Body Mass Index; *WC* waist circumference; *WHR* waist-to-hip ratio; *WHtR* waist-to-height ratio

### Subgroup analyses

In subgroup analyses fully stratified, it demonstrated a consistently significant positive relationship between TyG-BMI, TyG-WC, TyG-WHR, TyG-WHtR and CAP in participants without obesity (Table 4).

**Table 4:** Interaction of TyG index, modified TyG index and CAP in adolescents with or without obesity

Variables	Without obesity (n = 406)		With obesity (n = 126)		P for interaction
	β (95%CI)	*P* value	β (95%CI)	*P* value	
TyG—index	2.19 (− 11.20 ~ 15.57)	0.711	− 0.22 (− 42.78 ~ 42.35)	0.985	0.208
TyG-BMI	0.33 (0.19 ~ 0.48)	< 0.001	0.26 (− 0.19 ~ 0.72)	0.132	0.166
TyG-WC	0.16 (0.12 ~ 0.21)	< 0.001	0.11 (− 0.04 ~ 0.26)	0.085	0.079
TyG-WHR	12.80 (5.11 ~ 20.49)	0.006	7.76 (− 23.55 ~ 39.06)	0.398	0.192
TyG-WHtR	20.31 (11.79 ~ 28.82)	< 0.001	18.24 (− 9.12 ~ 45.61)	0.103	0.132

*TyG* Triglyceride to glucose index; *BMI* Body Mass Index; *WC* waist circumference; *WHR* waist-to-hip ratio; *WHtR* waist-to-height ratio; Each stratification was fully adjusted

### Sensitivity analyses

The sensitivity analysis in the refined NAFLD cohort (n = 527) confirmed the persistent associations between TyG indices and CAP (Supplemental Table 1).

## Discussion

This study investigated the relationship between TyG-related indices and MAFLD assessed by CAP in adolescents. Modified TyG indices (TyG-BMI, TyG-WC, TyG-WHR, TyG-WHtR) showed stronger associations with CAP than the original TyG index, which lost significance in fully adjusted models. Notably, TyG-WC demonstrated the highest diagnostic accuracy (AUC = 0.923), while TyG-BMI and TyG-WHtR also excelled in MAFLD prediction. These findings highlight that TyG indices combined with adiposity measures (BMI, WC, WHR, WHtR) outperform the TyG index alone, particularly in non-obese adolescents.

Our findings are consistent with previous reports on the association between TyG index and its modified forms with the risk of NAFLD [11, 25, 28]. However, this study uniquely identified significant positive associations between TyG-BMI, TyG-WC, TyG-WHR, TyG-WHtR, and MAFLD even after comprehensive covariate adjustment. These results align with and expand upon prior work by Wang et al.[13] (n = 1,171) showing TyG-BMI correlation with steatosis severity, while providing new evidence that TyG-WC demonstrates superior diagnostic performance (AUC = 0.923) compared to other indices, consistent with Song et al.'s observations in adults [26]. Our findings further corroborate Mendelian randomization evidence linking WHR to NAFLD risk [20],while novelly demonstrating the particular utility of TyG-WHtR (AUC = 0.915) in adolescent MAFLD detection, building upon prior observations in T2DM populations [29]. The significantly greater discriminative accuracy of modified indices (AUC range: 0.915–0.923) versus the original TyG index (AUC = 0.673; P < 0.001 for all comparisons) in our ROC analyses provides compelling evidence for incorporating anthropometric measures into TyG-based MAFLD screening tools.

The superior predictive performance of adiposity-enhanced TyG indices (TyG-BMI, TyG-WC, TyG-WHR, TyG-WHtR) compared to the conventional TyG index can be explained through several interrelated physiological mechanisms. First, these composite indices integrate both metabolic dysfunction (captured by the TyG component) and body fat distribution patterns (reflected in WC, WHR and WHtR), thereby providing a more comprehensive assessment of NAFLD risk factors. Importantly, WC serves as a direct marker of visceral adiposity [30], which is mechanistically linked to NAFLD pathogenesis through increased free fatty acid delivery to the liver and adipokine dysregulation. Furthermore, WHR and WHtR offer distinct advantages over BMI by specifically quantifying central obesity [31, 32], a metabolic phenotype strongly associated with NAFLD development and progression [33]. The enhanced diagnostic accuracy of these modified indices likely stems from their ability to simultaneously evaluate two fundamental NAFLD pathways: 1) insulin resistance (represented by the TyG index) and 2) dysfunctional adipose tissue distribution and physiology (represented by the anthropometric measures). This dual-pathway assessment is supported by accumulating evidence that combined indices outperform standalone measures in detecting hepatic steatosis [13, 25, 26, 29], reflecting the multifactorial nature of NAFLD pathogenesis that involves both metabolic derangements and body composition abnormalities [34, 35].

Subgroup analyses revealed robust associations between adiposity-enhanced TyG indices (TyG-BMI, TyG-WC, TyG-WHR, TyG-WHtR) and hepatic steatosis (CAP) in non-obese adolescents, a finding that aligns with emerging evidence from multiple study designs. Our results corroborate the work of Li et al.[36] and extend the observations of Naoya et al.[37], whose ultrasound-based study in non-obese adults similarly identified TyG-BMI as a strong NAFLD/MAFLD predictor. Additionally, a Chinese longitudinal study with a 5-year follow up involving 841 individuals found that an increase in TyG-BMI were associated with a higher incidence of NAFLD, as confirmed by ultrasound [38]. Mechanistically, while non-obese MAFLD shares pathophysiological features with obese MAFLD [39], genetic studies suggest unique modifiers like GCKR variants may influence WC-associated risk in lean individuals [40]. These findings underscore the importance of screening non-obese individuals with elevated modified TyG indices for NAFLD, as they may represent a high-risk subgroup despite their normal BMI.

Our study has several limitations that should be acknowledged. First, the cross-sectional design prevents determination of causal relationships between modified TyG indices and NAFLD, necessitating future prospective cohort studies for validation. Second, while transient elastography with CAP provides reliable non-invasive steatosis assessment, the lack of liver biopsy confirmation (the diagnostic gold standard) may affect diagnostic precision. Third, although NHANES'complex sampling design with oversampling of minority groups and post-stratification weighting ensures nationally representative estimates, external validation in independent pediatric cohorts would strengthen our findings. Notwithstanding these limitations, our study possesses important strengths: (1) use of a nationally representative sample with rigorous sampling methodology enhances generalizability; (2) employment of standardized CAP measurements provides objective, quantitative steatosis assessment superior to conventional ultrasound; and (3) comprehensive analytical approaches including multivariable regression and restricted cubic spline analyses strengthen the validity of our conclusions.

## Conclusion

In conclusion, modified TyG indices (TyG-BMI, TyG-WC, TyG-WHR, TyG-WHtR) exhibit stronger associations with MAFLD than the original TyG index in adolescents, particularly in non-obese individuals. These indices not only maintained significant correlations with CAP after full adjustment but also showed superior diagnostic accuracy (AUCs 0.915–0.923), with TyG-WC performing best (AUC = 0.923). These results highlight the clinical utility of incorporating anthropometric parameters (BMI, WC, WHR, WHtR) with the TyG index to improve MAFLD risk stratification in pediatric populations, offering a practical, non-invasive screening approach.

## Data Availability

All the data are available and can be freely downloaded from the National Health and Nutrition Examination Survey dataset (https://www.cdc.gov/nchs/nhanes/index.htm).

## Abbreviations

MAFLD: Metabolic associated fatty liver disease
NAFLD: Non-alcoholic fatty liver disease
NASH: Non-alcoholic steatohepatitis
HCC: Hepatocellular carcinoma
IR: Insulin resistance
TyG index: Triglyceride-glucose index
BMI: Body mass index
WC: Waist circumference
WHR: Waist-to-hip ratio
WHtR: Waist-to-height ratio
NHANES: National health and nutrition examination survey
NCHS: National center for health statistics
MEC: Mobile examination center
CAP: Controlled attenuation parameters
VCTE: Vibration-controlled transient elastography
RCS: Restricted spline regression
AST: Aspartate aminotransferase
ALT: Alanine aminotransferase
HbA1c: Glycohemoglobin
TC: Total cholesterol
LDL: Low-density lipoprotein
HDL: High-density lipoprotein
CRP: C-reactive protein
ROC: Receiver operating characteristic
AUC: Area under the curve

## References

[1] Amini-Salehi E, Letafatkar N, Norouzi N, Joukar F, Habibi A, Javid M, et al. Global prevalence of nonalcoholic fatty liver disease: an updated review meta-analysis comprising a population of 78 million from 38 countries. Arch Med Res. 2024;55: 103043. 10.1016/j.arcmed.2024.103043. 39094335 10.1016/j.arcmed.2024.103043

[2] Lee EJ, Choi M, Ahn SB, Yoo J-J, Kang SH, Cho Y, et al. Prevalence of nonalcoholic fatty liver disease in pediatrics and adolescents: a systematic review and meta-analysis. World J Pediatr. 2024;20:569–80. 10.1007/s12519-024-00814-1. 38771552 10.1007/s12519-024-00814-1

[3] Bardugo A, Bendor CD, Zucker I, Lutski M, Cukierman-Yaffe T, Derazne E, et al. Adolescent nonalcoholic fatty liver disease and type 2 diabetes in young adulthood. J Clin Endocrinol Metab. 2021;106:e34-44. 10.1210/clinem/dgaa753. 33075820 10.1210/clinem/dgaa753

[4] Lv J, Zhang Y, Li X, Guo H, Yang C. The burden of non-alcoholic fatty liver disease among working-age people in the Western Pacific Region, 1990–2019: an age–period–cohort analysis of the Global Burden of Disease study. BMC Public Health. 2024;24:1852. 10.1186/s12889-024-19047-y. 38992625 10.1186/s12889-024-19047-y PMC11238482

[5] Eslam M, Newsome PN, Sarin SK, Anstee QM, Targher G, Romero-Gomez M, et al. A new definition for metabolic dysfunction-associated fatty liver disease: An international expert consensus statement. J Hepatol. 2020;73:202–9. 10.1016/j.jhep.2020.03.039. 32278004 10.1016/j.jhep.2020.03.039

[6] Lim GEH, Tang A, Ng CH, Chin YH, Lim WH, Tan DJH, et al. An observational data meta-analysis on the differences in prevalence and risk factors between MAFLD vs NAFLD. Clin Gastroenterol Hepatol. 2023;21:619-629.e7. 10.1016/j.cgh.2021.11.038. 34871813 10.1016/j.cgh.2021.11.038

[7] Liu J, Ayada I, Zhang X, Wang L, Li Y, Wen T, et al. Estimating global prevalence of metabolic dysfunction-associated fatty liver disease in overweight or obese adults. Clin Gastroenterol Hepatol. 2022;20:e573–82. 10.1016/j.cgh.2021.02.030. 33618024 10.1016/j.cgh.2021.02.030

[8] Fang Y-L, Chen H, Wang C-L, Liang L. Pathogenesis of non-alcoholic fatty liver disease in children and adolescence: From “two hit theory” to “multiple hit model.” WJG. 2018;24:2974–83. 10.3748/wjg.v24.i27.2974. 30038464 10.3748/wjg.v24.i27.2974 PMC6054950

[9] Pal SC, Méndez-Sánchez N. Insulin resistance and adipose tissue interactions as the cornerstone of metabolic (dysfunction)-associated fatty liver disease pathogenesis. World J Gastroenterol. 2023;29:3999–4008. 10.3748/wjg.v29.i25.3999. 37476582 10.3748/wjg.v29.i25.3999 PMC10354585

[10] Sun Y, Ji H, Sun W, An X, Lian F. Triglyceride glucose (TyG) index: A promising biomarker for diagnosis and treatment of different diseases. Eur J Intern Med. 2024;:S0953620524003753. 10.1016/j.ejim.2024.08.026. 10.1016/j.ejim.2024.08.026 39510865

[11] Zhang S, Du T, Zhang J, Lu H, Lin X, Xie J, et al. The triglyceride and glucose index (TyG) is an effective biomarker to identify nonalcoholic fatty liver disease. Lipids Health Dis. 2017;16:15. 10.1186/s12944-017-0409-6. 28103934 10.1186/s12944-017-0409-6 PMC5248473

[12] Feng X, Deng Y, Chen C, Liu X, Huang Y, Feng Y. Predictive value of triglyceride-glucose index for all-cause and cardiovascular mortality in patients with diabetes mellitus: a retrospective study. Int J Endocrinol. 2024;2024: 6417205. 10.1155/2024/6417205. 39479579 10.1155/2024/6417205 PMC11524704

[13] Wang M, Chang M, Shen P, Wei W, Li H, Shen G. Application value of triglyceride-glucose index and triglyceride-glucose body mass index in evaluating the degree of hepatic steatosis in non-alcoholic fatty liver disease. Lipids Health Dis. 2023;22:186. 10.1186/s12944-023-01954-5. 37924128 10.1186/s12944-023-01954-5 PMC10623715

[14] Zeng P, Cai X, Yu X, Gong L. Markers of insulin resistance associated with non-alcoholic fatty liver disease in non-diabetic population. Sci Rep. 2023;13:20470. 10.1038/s41598-023-47269-4. 37993481 10.1038/s41598-023-47269-4 PMC10665395

[15] Liu H, Chen J, Qin Q, Yan S, Wang Y, Li J, et al. Association between TyG index trajectory and new-onset lean NAFLD: a longitudinal study. Front Endocrinol. 2024;15: 1321922. 10.3389/fendo.2024.1321922. 10.3389/fendo.2024.1321922 PMC10927994 38476672

[16] Gu Z, Li D, He H, Wang J, Hu X, Zhang P, et al. Body mass index, waist circumference, and waist-to-height ratio for prediction of multiple metabolic risk factors in Chinese elderly population. Sci Rep. 2018;8:385. 10.1038/s41598-017-18854-1. 29321674 10.1038/s41598-017-18854-1 PMC5762873

[17] Lee S, Kuk JL, Boesch C, Arslanian S. Waist circumference is associated with liver fat in black and white adolescents. Appl Physiol Nutr Metab. 2017;42:829–33. 10.1139/apnm-2016-0410. 28334548 10.1139/apnm-2016-0410

[18] Lin M-S, Lin T-H, Guo S-E, Tsai M-H, Chiang M-S, Huang T-J, et al. Waist-to-height ratio is a useful index for nonalcoholic fatty liver disease in children and adolescents: a secondary data analysis. BMC Public Health. 2017;17:851. 10.1186/s12889-017-4868-5. 29084519 10.1186/s12889-017-4868-5 PMC5663116

[19] Cai J, Lin C, Lai S, Liu Y, Liang M, Qin Y, et al. Waist-to-height ratio, an optimal anthropometric indicator for metabolic dysfunction associated fatty liver disease in the Western Chinese male population. Lipids Health Dis. 2021;20:145. 10.1186/s12944-021-01568-9. 34706716 10.1186/s12944-021-01568-9 PMC8549212

[20] Xie W, Hong Y, Chen X, Wang S, Zhang F, Chi X. Waist-to-hip ratio and nonalcoholic fatty liver disease: a clinical observational and Mendelian randomization analysis. Front Nutr. 2024;11: 1426749. 10.3389/fnut.2024.1426749. 39555187 10.3389/fnut.2024.1426749 PMC11563977

[21] Borges-Canha M, Neves J, Silva M, Mendonça F, Moreno T, Ribeiro S, et al. Waist-to-Hip ratio and inflammatory parameters are associated with risk of non-alcoholic fatty liver disease in patients with morbid obesity. Biomedicines. 2022;10: 2416. 10.3390/biomedicines10102416. 36289677 10.3390/biomedicines10102416 PMC9598594

[22] Karlas T, Petroff D, Sasso M, Fan J-G, Mi Y-Q, De Lédinghen V, et al. Individual patient data meta-analysis of controlled attenuation parameter (CAP) technology for assessing steatosis. J Hepatol. 2017;66:1022–30. 10.1016/j.jhep.2016.12.022. 28039099 10.1016/j.jhep.2016.12.022

[23] Eslam M, Alkhouri N, Vajro P, Baumann U, Weiss R, Socha P, et al. Defining paediatric metabolic (dysfunction)-associated fatty liver disease: an international expert consensus statement. Lancet Gastroenterol Hepatol. 2021;6:864–73. 10.1016/S2468-1253(21)00183-7. 34364544 10.1016/S2468-1253(21)00183-7

[24] Simental-Mendía LE, Gamboa-Gómez CI, Aradillas-García C, Rodríguez-Morán M, Guerrero-Romero F. The triglyceride and glucose index is a useful biomarker to recognize glucose disorders in apparently healthy children and adolescents. Eur J Pediatr. 2020;179:953–8. 10.1007/s00431-020-03570-2. 32016604 10.1007/s00431-020-03570-2

[25] Song K, Lee HW, Choi HS, Park G, Lee HS, Kim SJ, et al. Comparison of the modified TyG indices and other parameters to predict non-alcoholic fatty liver disease in youth. Biology. 2022;11: 685. 10.3390/biology11050685. 35625413 10.3390/biology11050685 PMC9138077

[26] Song S, Son D-H, Baik S-J, Cho W-J, Lee Y-J. Triglyceride glucose-waist circumference (TyG-WC) is a reliable marker to predict non-alcoholic fatty liver disease. Biomedicines. 2022;10: 2251. 10.3390/biomedicines10092251. 36140352 10.3390/biomedicines10092251 PMC9496145

[27] ElSayed NA, Aleppo G, Aroda VR, Bannuru RR, Brown FM, Bruemmer D, et al. 2. Classification and diagnosis of diabetes: standards of care in diabetes—2023. Diabetes Care. 2023;46 Supplement_1:S19-40. 10.2337/dc23-S002. 36507649 10.2337/dc23-S002 PMC9810477

[28] Ning Q, Zheng K, Yan J, Zhu C. Triglyceride glucose index as a predictor for non-alcoholic fatty liver disease: insights from a longitudinal analysis in non-obese individuals. Front Med (Lausanne). 2024;11: 1429413. 10.3389/fmed.2024.1429413. 39040897 10.3389/fmed.2024.1429413 PMC11260781

[29] Malek M, Khamseh ME, Chehrehgosha H, Nobarani S, Alaei-Shahmiri F. Triglyceride glucose-waist to height ratio: a novel and effective marker for identifying hepatic steatosis in individuals with type 2 diabetes mellitus. Endocrine. 2021;74:538–45. 10.1007/s12020-021-02815-w. 34355342 10.1007/s12020-021-02815-w

[30] Lee J-H, Jeon S, Lee HS, Kwon Y-J. Association between waist circumference trajectories and incident non-alcoholic fatty liver disease. Obes Res Clin Pract. 2023;17:398–404. 10.1016/j.orcp.2023.09.005. 37704496 10.1016/j.orcp.2023.09.005

[31] Pimenta NM, Santa-Clara H, Melo X, Cortez-Pinto H, Silva-Nunes J, Sardinha LB. Waist-to-hip ratio is related to body fat content and distribution regardless of the waist circumference measurement protocol in nonalcoholic fatty liver disease patients. Int J Sport Nutr Exerc Metab. 2016;26:307–14. 10.1123/ijsnem.2014-0256. 26630411 10.1123/ijsnem.2014-0256

[32] Pimenta NM, Cortez-Pinto H, Melo X, Silva-Nunes J, Sardinha LB, Santa-Clara H. Waist-to-height ratio is independently related to whole and central body fat, regardless of the waist circumference measurement protocol, in non-alcoholic fatty liver disease patients. J Human Nutr Diet. 2017;30:185–92. 10.1111/jhn.12410. 27600326 10.1111/jhn.12410

[33] Ismaiel A, Hosiny BE, Ismaiel M, Leucuta D-C, Popa S-L, Catana CS, et al. Waist to height ratio in nonalcoholic fatty liver disease - Systematic review and meta-analysis. Clin Res Hepatol Gastroenterol. 2023;47: 102160. 10.1016/j.clinre.2023.102160. 37321322 10.1016/j.clinre.2023.102160

[34] Liu X, Tang Y, Luo Y, Gao Y, He L. Role and mechanism of specialized pro-resolving mediators in obesity-associated insulin resistance. Lipids Health Dis. 2024;23:234. 10.1186/s12944-024-02207-9. 39080624 10.1186/s12944-024-02207-9 PMC11290132

[35] Fu Y, Hua Y, Alam N, Liu E. Progress in the study of animal models of metabolic dysfunction-associated steatotic liver disease. Nutrients. 2024;16: 3120. 10.3390/nu16183120. 39339720 10.3390/nu16183120 PMC11435380

[36] Li S, Feng L, Ding J, Zhou W, Yuan T, Mao J. Triglyceride glucose-waist circumference: the optimum index to screen nonalcoholic fatty liver disease in non-obese adults. BMC Gastroenterol. 2023;23:376. 10.1186/s12876-023-03007-8. 37919650 10.1186/s12876-023-03007-8 PMC10621119

[37] Otsubo N, Fukuda T, Cho G, Ishibashi F, Yamada T, Monzen K. Utility of indices obtained during medical checkups for predicting fatty liver disease in non-obese people. Intern Med. 2023;62:2307–19. 10.2169/internalmedicine.1097-22. 36517035 10.2169/internalmedicine.1097-22 PMC10484762

[38] Li Y, Zheng R, Li J, Feng S, Wang L, Huang Z. Association between triglyceride glucose-body mass index and non-alcoholic fatty liver disease in the non-obese Chinese population with normal blood lipid levels: a secondary analysis based on a prospective cohort study. Lipids Health Dis. 2020;19:229. 10.1186/s12944-020-01409-1. 33109219 10.1186/s12944-020-01409-1 PMC7592551

[39] Phipps M, Wattacheril J. Non-alcoholic fatty liver disease (NAFLD) in non-obese individuals. Frontline Gastroenterol. 2020;11:478–83. 10.1136/flgastro-2018-101119. 33101626 10.1136/flgastro-2018-101119 PMC7569516

[40] Wu N, Li J, Zhang J, Yuan F, Yu N, Zhang F, et al. Waist circumference mediates the association between rs1260326 in GCKR gene and the odds of lean NAFLD. Sci Rep. 2023;13:6488. 10.1038/s41598-023-33753-4. 37081070 10.1038/s41598-023-33753-4 PMC10119110

